# Carotenoids: Dietary Sources, Extraction, Encapsulation, Bioavailability, and Health Benefits—A Review of Recent Advancements

**DOI:** 10.3390/antiox11040795

**Published:** 2022-04-18

**Authors:** Ramesh Kumar Saini, Parchuri Prasad, Veeresh Lokesh, Xiaomin Shang, Juhyun Shin, Young-Soo Keum, Ji-Ho Lee

**Affiliations:** 1 Department of Crop Science, Konkuk University, Seoul 05029, Korea; saini1997@konkuk.ac.kr (R.K.S.); rational@konkuk.ac.kr (Y.-S.K.); 2 Institute of Biological Chemistry, Washington State University, Pullman, WA 99164, USA; prasad.parchuri@wsu.edu; 3 Biocontrol Laboratory, University of Horticultural Sciences, Bagalkote 587104, India; loksv.biotech@gmail.com; 4 Jilin Provincial Key Laboratory of Nutrition and Functional Food, Jilin University, Changchun 130062, China; xmshang@jlu.edu.cn; 5 Department of Stem Cell and Regenerative Biotechnology, Konkuk University, Seoul 05029, Korea; junejhs@konkuk.ac.kr

**Keywords:** astaxanthin, β-carotene, cardiovascular diseases (CVDs), cancer, diabetes, lycopene, microalgae, neurodegenerative disease, pigments, vitamin A

## Abstract

Natural carotenoids (CARs), viz. β-carotene, lutein, astaxanthin, bixin, norbixin, capsanthin, lycopene, canthaxanthin, β-Apo-8-carotenal, zeaxanthin, and β-apo-8-carotenal-ester, are being studied as potential candidates in fields such as food, feed, nutraceuticals, and cosmeceuticals. CAR research is advancing in the following three major fields: (1) CAR production from natural sources and optimization of its downstream processing; (2) encapsulation for enhanced physical and chemical properties; and (3) preclinical, clinical, and epidemiological studies of CARs’ health benefits. This review critically discusses the recent developments in studies of the chemistry and antioxidant activity, marketing trends, dietary sources, extraction, bioaccessibility and bioavailability, encapsulation methods, dietary intake, and health benefits of CARs. Preclinical, clinical, and epidemiological studies on cancer, obesity, type 2 diabetes (T2D), cardiovascular diseases (CVD), osteoporosis, neurodegenerative disease, mental health, eye, and skin health are also discussed.

## 1. Introduction

Clinical and epidemiological studies have evidenced an inverse association between dietary intake of fruits and vegetables and the incidence of chronic diseases such as type 2 diabetes (T2D), cardiovascular diseases (CVDs), and cancer, as well as all-cause mortality [1,2,3,4]. The bioactive antioxidant compounds, including carotenoids (CARs), present in fruits and vegetables detoxify the free radicals in the cells, thus minimizing oxidative damage and incidence of related diseases [5,6,7,8,9,10,11].

CARs are red, yellow, and orange tetraterpenoid pigments universally synthesized by all terrestrial and aquatic photoautotrophs, including plants, microalgae, and macroalgae. Interestingly, some nonphotosynthetic bacteria, some insects (pea aphids, spider mites, and some species of gall midges), and some fungi can also synthesize CARs [12]. In microalgae and plant cells, CARs are biosynthesized and stored in plastids, where they play essential roles in oxygenic photosynthesis (light harvesting), photoprotection (detoxification of free radicals generated during photosynthesis), and signaling pathways [13,14,15]. Moreover, in plants, CARs serve as a precursor to the biosynthesis of phytohormones such as abscisic acid and strigolactones, which play a crucial role in regulating several plant developmental and adaptation processes [16].

Plastids are crucial in controlling carotenogenic activity, pigment diversity, and CAR stability [13]. Moreover, cultivational, environmental (light intensity, drought, salinity, and chilling stresses), and genetic factors have shown to significantly influence the CAR contents of crop plants [17].

Apart from the central functions in plants and other photosynthetic microbes, CARs play essential roles by providing a dietary source of provitamin A (e.g., α- and β-carotene and β-cryptoxanthin). Moreover, the antioxidant activities of CARs regulate oxidative stress (stabilize cellular membranes) and inflammatory mediators, thus protecting against metabolic syndromes (MetS: CVD and T2D), cancer, neurodegenerative diseases, and photooxidative damage to the skin and eye [11,18].

As most animals are incapable of biosynthesizing CARs, they need to obtain CARs from their diets [19]. Moreover, intake of CARs can be enhanced by dietary supplements, which have seen exponential growth in recent years. For example, several CAR-based formulations for sports nutrition (physical stamina), improved bone health, antiaging, and vision are available in the market [20,21,22,23]. Moreover, CARs are substantially used in aquatic animals and poultry feed. Applications of CARs in poultry feed improve the pigmentation of egg yolk, a symbol of eggs with a high marketing quality [21]. In addition to egg yolk pigmentation, CAR supplementation in poultry improves the health of prehatched and posthatched birds via oxidative stress regulation [21]. In addition, for better pigmentation (marketing quality) and improved health and reproductive performance, astaxanthin is the most common supplement in the feed of salmon, trout, sea bream, and ornamental fishes [22,24].

The first chromatographic separation of CARs was achieved in 1906, and the structures of β-carotene and lycopene were elucidated in 1930 [25]. To date, 1204 CARs have been identified from natural sources, and these are mainly composed of 40-carbon chains (C40: 1121 CARs), followed by C50 (37), C30 (33), and C45 (13) [26]. The simple hydrocarbon CARs are known as carotenes (e.g., lycopene and α- and β-carotene), and the oxygenated derivatives of carotenes are termed xanthophylls [12]. In xanthophylls, the presence of different oxygen-containing functional groups, such as hydroxyl (e.g., β-cryptoxanthin, lutein, and zeaxanthin), carbonyl (e.g., capsanthin, astaxanthin, and canthaxanthin), epoxide (e.g., violaxanthin, neoxanthin, and fucoxanthin), or many other functional groups, contribute to the diversity of CARs [23,27]. The simple hydrocarbon structure of carotenes makes them nonpolar, while the xanthophylls are polar molecules.

This review critically discusses the chemistry and antioxidant activity, marketing trends, dietary sources, extraction, bioaccessibility and bioavailability, encapsulation methods, intake (normal, safe, and desirable), and health benefits of CARs. Epidemiological, clinical, and preclinical studies on cancer, obesity, T2D, CVDs, osteoporosis, neurodegenerative disease, mental health, eye, and skin health will also be discussed.

## 2. Chemistry and Antioxidant Activity of CARs

**Figure 1:**
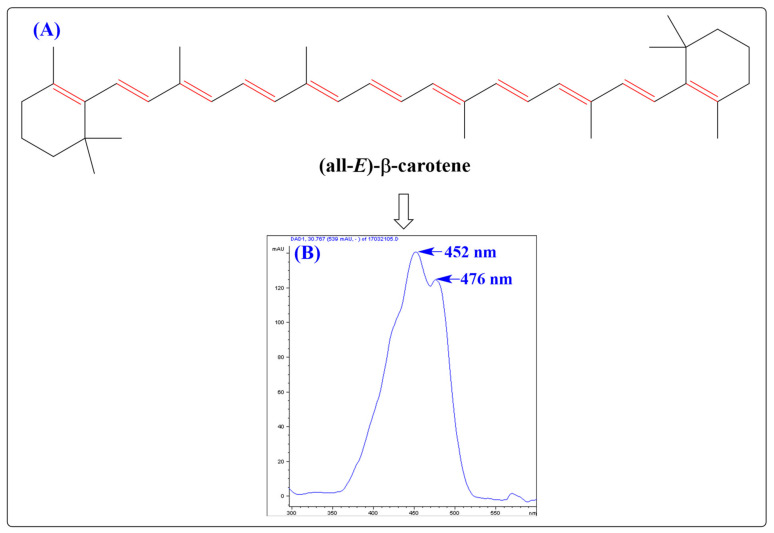
The molecular structure of chromophore of (all-*E*)-β-carotene (**A**), responsible for the absorption of light in the visible range. The absorbance spectrum of (all-*E*)-β-carotene (**B**) is from carrots recorded using a diode array detector (DAD) in the solvent system previously used in our study [29] (unpublished data).

Most CARs are derivatives of the lipid-soluble tetraterpenoid C40 pigment (15*Z*)-phytoene (a colorless intermediate in the biosynthesis of CARs) biosynthesized from the C_5_ isoprenoid precursor’s dimethylallyl pyrophosphate (DMAPP) and its allylic isomer isopentenyl pyrophosphate (IPP) [28]. The long polyene chain (–C=C–) structure with 8–13 conjugated double bonds forms the chromophore of the CAR molecule, responsible for their coloration/pigmentation properties (absorption of light in the visible range (400–500 nm) of the electromagnetic spectrum) [12] (Figure 1). Moreover, this long polyene chain structure delivers a resonance-stabilized intermediate for efficient quenching of reactive oxygen species (ROS).

The length of the polyene chain, the presence of functional groups and their positioning (e.g., α and β), glycosylation of a hydroxyl group, esterification with fatty acids, acyclic and cyclic structure, and (*E*)- or (*Z*)-configuration are known to significantly affect the antioxidant and biological properties of CARs, including their anticancer potential [28,30,31,32]. In vitro studies have suggested that the monocyclic structure of a CAR is more favorable for singlet oxygen (^1^O_2_) quenching than an acyclic structure [31]. Moreover, CARs with keto functional groups (especially at C-4/C-4′; e.g., canthaxanthin) possess a higher antioxidant potential than hydroxy derivatives (e.g., zeaxanthin), probably due to the extended conjugated double-bond system (13 in canthaxanthin vs. 11 in zeaxanthin) [31].

The (*Z*)-isomers of lycopene and astaxanthin have shown greater bioavailability and bioactivity than the (all-*E*)-isomers [33,34]. The (*Z*)-isomerization of (all-*E*)-CARs is mainly achieved by light irradiation, heat, and catalytic treatments. Moreover, in recent years, natural catalysts, such as isothiocyanates and polysulfides naturally present in mustard, onion, and garlic, have been utilized in the (Z)-isomerization of lycopene, β-carotene, and astaxanthin [34]. In addition, the (*Z*)-isomerization efficiency of these natural catalysts can be substantially improved by the addition of the synthetic antioxidants butylated hydroxytoluene (BHT) or α-tocopherol, as these antioxidants minimize the thermal degradation of CARs at the prolonged (≈1 h) high reaction temperature of 60–80 °C [34]. 

In contrast to the resonance-stabilized intermediate for efficient quenching of ROS, the unsaturated polyene chain structure of CAR is prone to oxidation, hydrolysis, isomerization, and degradation mediated by heat, light, oxygen, catalysts, and other factors [35,36,37]. In general, carotenes are more prone to thermal degradation than xanthophylls. The degradation rates of (all-*E*)-lutein, zeaxanthin, β-cryptoxanthin, and β-carotene at 25 and 35 °C (with iodine (1–2 wt % in hexane) and 1800 lx light) were described by a first-order kinetic model, in the order of β-carotene > β-cryptoxanthin > lutein > zeaxanthin [35]. In this study, it was shown that the degradation of these CARs produced several mono-(*Z*)-isomers, di-(*Z*)-isomers, and oxidation products.

Among astaxanthin, meso-zeaxanthin, fucoxanthin, and β-carotene, meso-zeaxanthin and β-carotene exhibit the slowest photo-oxidation under ultraviolet (UV)–visible light/hydrogen peroxide (H_2_O_2_: source of hydroxyl radical (•OH), and other radicals), while fucoxanthin’s degradation is the fastest of all studied CARs, followed by astaxanthin [37], which suggests the excellent •OH scavenging properties of fucoxanthin and astaxanthin.

The oxidation of electron-rich CARs mediated by free radicals or other oxidizing agents (electrophilic reagents) leads to the formation of CAR peroxides that can substantially influence biological properties [37,38]. Thus, it has been suggested that any carotenoid used in biological studies must be free from peroxides, and preferably freshly isolated before the experiment, as storing samples in a freezer may also lead to bleaching and degradation [38].

**Figure 2:**
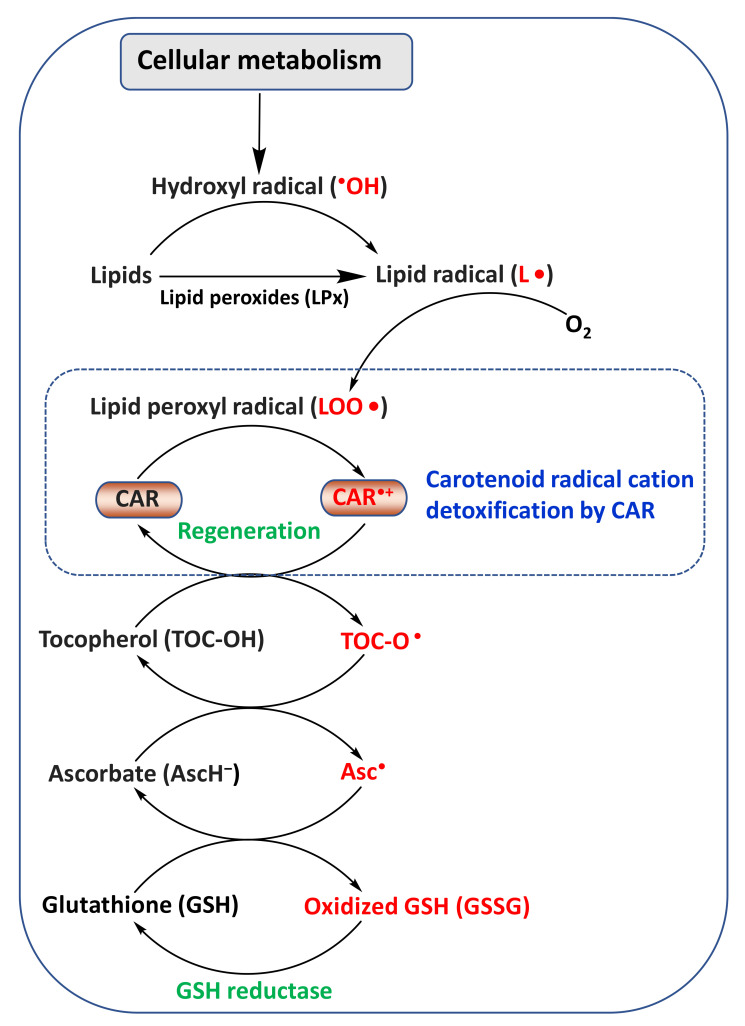
The lipid peroxyl radical (LOO•) scavenging/detoxification by carotenoids (CARs). The carotenoid radical cation (CAR•+) can be regenerated in the presence of tocopherol (vitamin E), ascorbate (vitamin C), and glutathione.

In general, ROS scavenging by CARs occurs by the following mechanisms: (i) radical adduct formation (ROO-CAR^•^); (ii) electron transfer between peroxyl radicals (ROO^•^) and CARs, leading to the formation of a CAR radical anion (CAR^•−^) and radical cation (CAR^•+^); and (iii) hydrogen atom transfer (HAT), which leads to a neutral-resonance-stabilized CAR radical (CAR^•^) [32,39]. CAR^•+^ can be regenerated to parent CAR by other cellular antioxidants (reducing agents), such as ascorbate (vitamin C), tocopherol (vitamin E), and glutathione [40,41,42] (Figure 2). Thus, it has been suggested that the detrimental effects of CAR^•+^ on human health can be minimized with appropriate levels of these reducing agents.

## 3. Marketing Trends of CARs

In 2019, the global market value of CARs was estimated at approximately USD 1.44 billion, and is expected to reach USD 1.84 billion in 2027, with a compound annual growth rate (CAGR) of 3.4% (https://www.fortunebusinessinsights.com, accessed on 25 February 2022). The carotenoids market is driven by: (i) growing concerns of synthetic CAR over natural; (ii) an increased focus on the consumption of natural products; and (iii) an increased demand for cosmetics and nutraceuticals.

**Figure 3:**
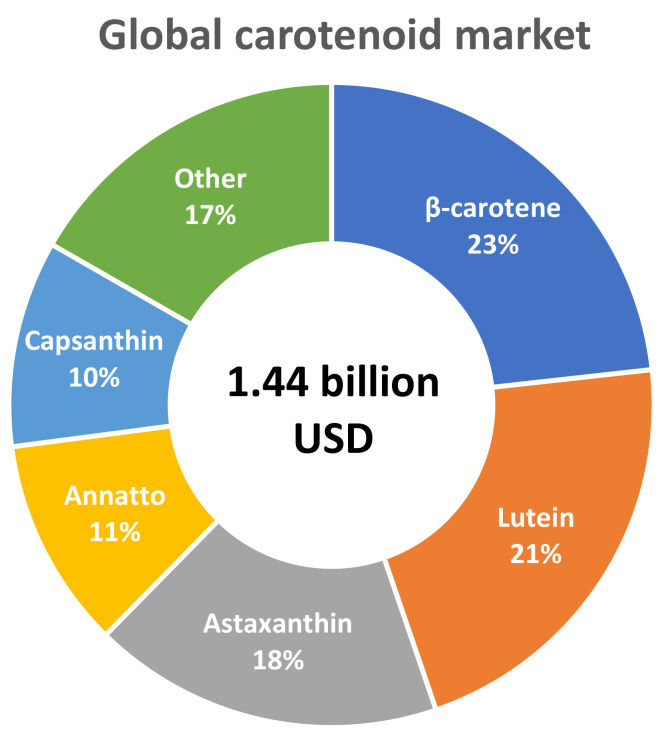
The marketing trends of carotenoids. Source: https://www.bccresearch.com, accessed on 25 February 2022.

Currently, the CAR market is dominated by β-carotene (23.2%), lutein (21.5%), astaxanthin (17.6%), annatto (10.6%; pigments from *Bixa orellana* L. seeds, composed of bixin and norbixin), capsanthin (10.4%), lycopene (5.85%), canthaxanthin, β-Apo-8-carotenal, zeaxanthin, and β-apo-8-carotenal-ester (https://www.bccresearch.com, accessed on 25 February 2022) (Figure 3). β-Carotene and astaxanthin are the CARs with the highest industrial production, and their global market potential is predicted to increase up to USD 520 million and USD 800 million by 2025, respectively [43,44]. Nearly 46% of the CARs market comprises animal feed, while food and beverages, dietary supplements, and cosmetics account for the rest.

## 4. Source of Natural CARs

**Figure 4:**
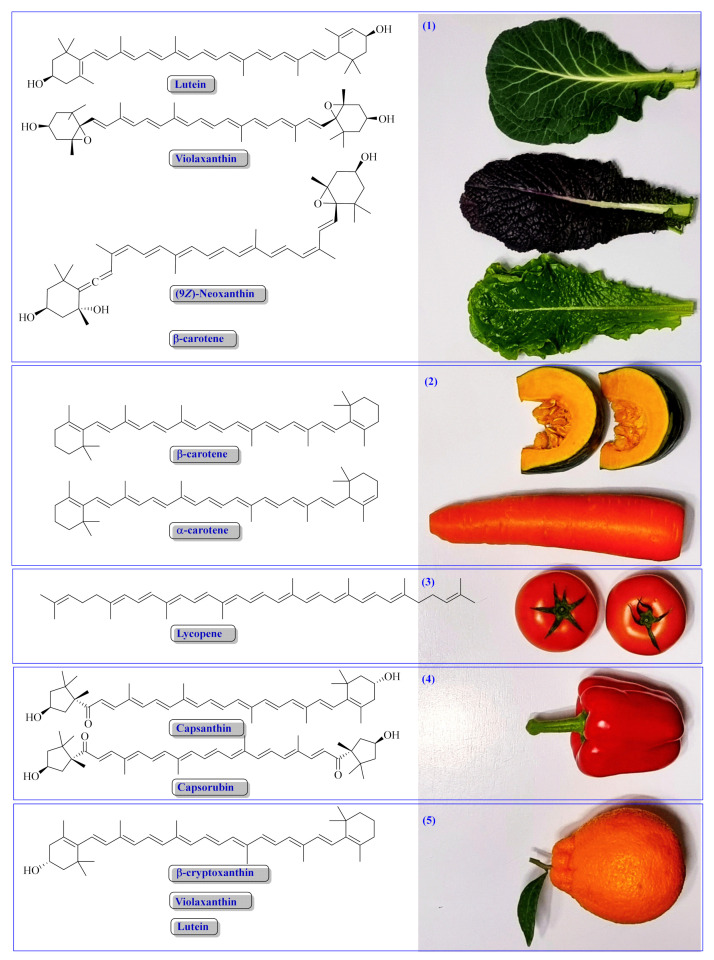
The dietary carotenoids obtained from the major fruits and vegetables. From top to bottom, (**1**) green leafy vegetables, (**2**) pumpkin and carrot, (**3**) tomatoes, (**4**) red paprika, and (**5**) orange.

Colored fruits and vegetables are the major dietary source of CARs in the human diet [45] (Figure 4). Moreover, considering their health-beneficial properties, dietary supplements can increase carotenoid intake. Currently, several provitamin A (e.g., β-carotene) and non-provitamin A (e.g., lutein and zeaxanthin for eye health)-based dietary supplements and many other products are available, produced and marketed mainly by BASF (Ludwigshafen, Germany), Chrysantis Inc. (West Chicago, IL, USA), DSM (Heerlen, The Netherlands), LycoRed Ltd. (Be’er Sheva, Israel), and OmniActive (Mumbai, India) [28,46,47].

### 4.1. Fruits, Vegetables, Grains and Other Higher Plant-Based Products

Among plant-based products, gac fruit (*Momordica cochinchinensis* (Lour.) Spreng.) arils are probably the richest sources of (all-*E*)-lycopene (164.4 mg/100 g FW) [45]. A high amount of lycopene was also recorded in the bitter melon (*Momordica charantia* L.) seed arils (27.3 mg/100 g fresh weight (FW)), accounting for 75% of the total CARs [48].

In the United States Department of Agriculture (USDA) Food and Nutrient Database, dried tomatoes are listed as the richest source of lycopene (46 mg/100 g of lycopene) (https://fdc.nal.usda.gov/fdc-app.html#/?component=1122, accessed on 15 February 2022), probably because gac fruit is not listed in this database. Due to their very high lycopene content and substantial consumption, tomato and tomato-based products such as sauce and ketchup are the most significant source of lycopene, especially in the United States. One serving (120 g) of tomato sauce provides ~7 mg of lycopene, and an average adult in the United States consumes 4.5 mg lycopene/d [49]. Other than in tomato, lycopene is substantially found in watermelon (1.6–3.5 mg/100 g FW), papaya (1.8–4.2 mg/100 g FW), and guava (3.2–7.0 mg/g100 g FW) [50].

β-Carotene is a dominating CAR in carrots (6.1–7.1 mg/100 g FW) [50], sweet potato, capsicum pods, and green leafy vegetables [23,28,51,52]. Lutein and β-carotene, followed by neoxanthin and violaxanthin, are the most dominant CARs of green leafy vegetables, for instance, 100 g of raw kale (FW basis) contains 5–6 mg lutein, 5–6 mg β-carotene, 1.2–2.3 mg neoxanthin, and 2.0–3.4 mg violaxanthin [29,50,51,52]. Among the green leafy vegetables, lactucaxanthin selectively occurs in lettuce (*Lactuca sativa* L.), with a high amount in romaine lettuce (*cv*. Super caesar red; 2.3 mg/100 g FW) [29,53].

Moreover, corn (*Zea mays* L.) seeds and egg yolk are good sources of lutein and zeaxanthin [54]. As corn forms >50% of laying-hen diets [55], it supplies lutein and zeaxanthin, which are responsible for the intense yellow-orange color of egg yolk. Lutein (0.714 mg/100 g) can also be found in dehulled black rice (*cv*. Sintoheugmi, Type—Japonica) [56]. Lutein is commonly extracted from marigold (*Tagetes* sp. L.) flower petals for commercial production [57]. 

Citrus, persimmon, peach, papaya fruits, and capsicum pods are a significant source of β-cryptoxanthin in the diet [49]. In most fruits, xanthophylls such as neoxanthin, lutein, zeaxanthin, and β-cryptoxanthin are predominately found in an esterified form (≈50–99% of total xanthophylls) [58]. In *Capsicum* pods, xanthophylls esters such as violaxanthin, lutein, zeaxanthin, and keto CARs (capsanthin, capsorubin diester) are predominantly found [58,59]. The total CAR contents of 14 (*cv*. Raon Red)–127 (*cv.* Mini Goggal Red) mg/100 g dry weight (DW) are recorded in red paprika (*Capsicum annuum* L.; bell pepper) pods of various cultivars, of which capsanthin accounts for ≈75%, except for *cv*. Mini Goggal Red, in which zeaxanthin accounts for 96% of the total CARs [60]. In addition, in orange paprika, zeaxanthin (89–151 mg/100 g DW) and lutein (17–28 mg/100 g DW) are dominant, with total CAR contents of 112–190 mg/100 g DW [60].

The stigmas of saffron (*Crocus sativus* L.), a popular food condiment, contain water-soluble carotenoids (e.g., crocetin esters), as well as apocarotenoids (e.g., picrocrocin and safranal), which are responsible for their color and aroma [61]. In saffron, the esterification of crocetins with sugars such as gentiobiose (G) and glucose (g) gives rise to the geometric isomers of crocin. In the hydroethanolic extract of Indian saffron, picrocrocin accounted for 18.1% (*w*/*w*), followed by (*E*)-4-gg-crocin (13.7%) and (*E*)-3-Gg-crocin (5.5%) [61].

### 4.2. Microalgae, Macroalgae (Seaweeds), and Fungi

Microalgae are being currently explored as a sustainable and alternative source in the feed (animal and aquaculture), food, nutraceutical, cosmeceutical, energy, and fine chemical industries [62,63,64]. Among the microalgae, *Dunaliella salina* (Dunal) Teodoresco and *Haematococcus pluvialis* Flotow are the richest sources of β-carotene and astaxanthin, respectively [28,62]. *D. salina* can accumulate up to 15% of β-carotene (dry cell weight (DCW), while *H. pluvialis* can accumulate up to 7% of astaxanthin (DCW); thus, these microalgae are widely used in the commercial production of β-carotene and astaxanthin, respectively [65,66,67,68,69,70]. 

In addition, commercial production using CAR-accumulating microalgae, such as canthaxanthin from *Chlorella zofingiensis* Dönz, lutein from *Scenedesmus* sp. Meyen, echinenone from *Botryococcus braunii* Kützing, and fucoxanthin from *Tisochrysis lutea* Bendif & Probert and *Phaeodactylum tricornutum* Bohlin, has been established [28,62,71,72]. The microalgae *Chlorella sorokiniana* Shihira & Krauss, *Parachlorella* sp. Krienitz, and *Desmodesmus* sp. (Chodat) An, Friedl & Hegewald also accumulate a good amount of lutein (5–12 mg/g DCW), and can be utilized in commercial production [65]. In view of their rapid growth rates and substantial amounts of fucoxanthin, microalgae such as *Isochrysis* sp. Parke and *Nitzschia* sp. Hassall may also be considered as a source of fucoxanthin for industrial production purposes, as studies have shown [72,73].

The conditions limiting photosynthesis or growth in the green stage, such as bright illumination, insufficient nitrogen, and salinity stress, are commonly adopted to trigger astaxanthin accumulation in *H. pluvialis* [24,74,75,76]. Moreover, in addition to abiotic stress, butylated hydroxyanisole (BHA), fulvic acid, melatonin, and polyamines (putrescine, spermidine, and spermine) are known to result in the accumulation of astaxanthin in *H. pluvialis* by regulating stress signals, carotenogenesis, and lipid metabolism pathway [74].

In addition to microalgae, the yeast *Phaffia rhodozyma* Mill., Yoney. & Soneda is a viable source for the industrial production of astaxanthin [77]. In addition, *Blakeslea trispora* Thaxt., a filamentous fungus, is used for the commercial production of β-carotene and lycopene [66,78]. Vitatene, a Spain-based maker of natural CARs, is producing commercial lycopene (LYCONAT^®^) and β-carotene (BETANAT^®^) from the fermentation of *B. trispora*; this company has been acquired by DSM (Heerlen, The Netherlands), a major market player that produces and markets natural and synthetic CARs (https://www.dsm.com, assessed on 7 January 2022). Currently, β-carotene obtained from *B. trispora* is marketed under the trade name CaroCare^®^ (https://www.maxihealth.com/view-catalog, assessed on 7 January 2022).

There is an emerging interest in the production of natural products. For example, Wu et al. successfully constructed microbial electrosynthesis (MES) systems by coupling de novo lycopene biosynthesis with water electrolysis, therefore producing lycopene with the valorization of CO_2_ as the carbon source [79]. However, substantial efforts are still required to optimize CAR metabolic pathways and the MES system to make such a production process feasible for commercial use.

Seaweeds (marine macroalgae), especially seaweeds belonging to Phaeophyceae (brown algae), are a unique source of fucoxanthin [80]. The marine seaweeds *Eisenia bicyclis* (Kjellman) Setchell; *Padina tetrastromatica* Hauck; *Sargassum fusiforme* (Harvey) Setch.; Saccharina japonica (Areschoug) Lane, Mayes, Druehl & Saunders; *Undaria pinnatifida* (Harvey) Suringar; *Himanthalia elongata* (L.) Gray; *Cystoseira hakodatensis* (Yendo) Fensholt; *Fucus vesiculosus* L.; and *Fucus serratus* L. are excellent sources of fucoxanthin [72,81].

### 4.3. Shellfish Species

Shellfish species (mollusks and crustaceans) are considered a key dietary source of health-beneficial omega-3 (n-3) long-chain polyunsaturated fatty acids (LC-PUFAs) eicosapentaenoic acid (EPA, C20:5), and docosahexaenoic acid (DHA, C22:6) [82,83], which play a crucial role in reducing the risk of CVDs [64,84]. Moreover, shellfish species are the major source of some microalgal CARs that are rarely found in plants. For instance, CARs such as astaxanthin are found in salmon and shrimp [85,86,87], echinenone in sea urchins; pectenoxanthin and pectenolone (an oxidative metabolite of diatoxanthin) in scallops [88]; peridinin, pyrrhoxanthin, and diadinoxanthin in tridacnid clam; and fucoxanthin and fucoxanthinol in *Modiolus modiolus* L. and *Pecten maximus* L. Interestingly, CAR accumulation in these species and other vertebrates (e.g., colorful birds and fish) occur in a gender-specific manner, suggesting that their biosynthesis may be regulated by sex hormones. 

### 4.4. Biofortified Crops and Microbes

Several CAR biofortified crops have been successfully developed using genetic modification (GM; transgenic or metabolic engineering) and breeding approaches [89,90,91,92]. In particular, CAR biofortification using plant breeding is considered to be a sustainable approach [93]. It helps to exploit the existing natural variation in CAR contents in plants by utilizing modern molecular breeding tools, such as targeting local induced lesions in genomes (TILLING) and marker-assisted selection (MAS) [91]. Maize hybrids developed through marker-assisted stacking of β-carotene biosynthetic genes (β-carotene hydroxylase, lycopene-ε-cyclase) showed a 4.5-fold increase in the concentration of provitamin A (β-carotene and β-cryptoxanthin) compared to original hybrids [93]. Several CAR-biofortified crops developed using breeding approaches have already been released worldwide [90], such as carotene-fortified wheat (variety HI 8627); cauliflower (Pusa Beta kesari variety); mango in India; provitamin-A-rich maize in Zambia, Nigeria, and Ghana; provitamin-A-rich cassava in Nigeria; and finally, provitamin-A-rich banana in Burundi [93]. 

These breeding methods are not applicable if the natural variation in CAR content is insufficient or not present in the target crop. Therefore, genetic-engineering approaches help to introduce CAR biosynthesis genes from non-native sources. β-Carotene biofortified rice (golden rice), first developed in the 1990s by GM and then modified in 2004, is a well-known example of successful GM of crop plants for CAR biofortification. Moreover, it could be an important public health intervention for β-carotene supplementation worldwide [94]. However, despite the consumption of golden rice being declared safe for human consumption by Australia, New Zealand, Canada, and the United States, and approved for commercial cultivation by the Philippines [95], commercial cultivation is still not approved in most countries, given the possible concerns regarding human or environmental health risks [94]. Several other GM crops enriched with CARs are successfully produced [91]; however, none of them are under commercial cultivation.

Microbial CARs are emerging as a low-cost alternative source of CARs, owing to the ability of microbes to utilize cheaper agroindustrial wastes as substrates during the fermentation process [44]. Several multigene metabolic pathways, including precursor biosynthesis by the 2-C-methyld-erythritol 4-phosphate (MEP) and mevalonate (MVA) pathways, the CARs biosynthetic pathway, and the apo CAR biosynthetic pathways, have been genetically engineered in microbes for CAR biofortification [28]. The introduction of precursor and CARs biosynthesis pathway from *Mucor circinelloides* Tiegh. into *Yarrowia lipolytica* (Wick., Kurtzman & Herman) Van der Walt & Arx by the clustered regularly interspaced short palindromic repeats (CRISPR)/CRISPR-associated protein 9 (Cas9) strategy resulted in a 24-fold higher accumulation of β-carotene (408 mg/L in shake flask cultures), compared to the parental strain *Y. lipolytica* XK2 [96].

## 5. Extraction of CARs: Pretreatments Improve the Recovery

The extraction recovery of CARs mainly depends on the characteristics of the extraction method’s parameters (e.g., solvent, pressure, and temperature) and food matrix (e.g., cell wall characteristics, carotenoid composition, and moisture content) [23]. Among these, selecting an appropriate solvent or solvent combination is one of the most vital factors in the efficient extraction of CARs. Usually, hexane and acetone are selected to extract nonpolar and polar CARs, respectively. On the other hand, a mixture of acetone/ethanol/hexane is commonly utilized for the simultaneous extraction of nonpolar and polar CARs [23].

Microwave-assisted extraction (MAE) [97], ultrasonication-assisted extraction (UAE) [98,99], and supercritical CO_2_ extraction (SCE) are emerging as extraction methods for CARs [68,87]. SCE is known as the best technique to extract CARs as dietary supplements, since the issue of residual toxicity from harmful solvents can be eliminated. However, SCE provides a low yield of polar CARs (xanthophylls), which can be improved by adding ethanol as a cosolvent [100]. A study using SCE of CARs from 15 different fruits and vegetables, including sweet potato (flesh and peels), tomato, apricot, pumpkin, peach, and paprika (green, yellow and red), utilizing the optimized conditions (59 °C, 350 bar, 15 g/min CO_2_, and 30 min of extraction time) with 15.5% (*v*/*v*) ethanol as a cosolvent, provided >90% recovery (*w*/*w*) [100].

UAE (40 kHz, power of 100 W, 50 °C, 30 min) utilizing n-hexane/acetone (3:1, *v*/*v*) applied to tomato peel obtained a CAR-rich extract with total CAR and lycopene contents of 261.74 and 166.71 mg/g DW, respectively [98]. The microencapsulation (using complex coacervation and freeze-drying) of this extract showed 63% lycopene retention after storage at 4 °C in the dark for 14 days.

Among ethanol, acetone, ethanol/acetone (1:1 *v*/*v*), and ethanol/acetone/hexane (1:1:2 *v*/*v*/*v*), which were utilized for the extraction of CARs from the β-cryptoxanthin-dominated (49.2% of total CARs) persimmon peel waste, the highest amount of total CARs (339 mg/g extract) was obtained using acetone, followed by ethanol (139 mg/g extract). At the same time, other solvents and solvent combinations provided substantially lower yields. Moreover, in this study, various in vitro methods that evaluated the antioxidant activities showed similar trends, with the highest activities in the acetone extract.

Plant and microalgal cells are composed of rigid cell walls, which inhibit the entry of solvents into the cell. In addition, a close association between CARs and other macromolecules, such as fatty acids and proteins, prevents the mass transfer of CARs during extraction. Thus, in the first step of extraction, these barriers are disrupted by physical (bead milling, high-pressure homogenization (HPH), hydrodynamic cavitation, cooking, osmotic shock, ultrasonication, microwave irradiation, cryogenic grinding, and pulsed electric field (PEF) application), chemical (acid, base, and surfactant treatments), or enzymatic or biological (e.g., germination of *H. pluvialis* cyst cell) means to improve the extraction of CARs [23,101,102]. The selection of an appropriate pretreatment method depends on the cellular matrix and cell-wall characteristics. For instance, the firm structure of trilayered cell walls in *H. pluvialis* requires extreme methods for efficient cell disruption [23,101].

MAE and UAE are commonly utilized to disrupt intermolecular forces and facilitate solvent penetration and extraction of bioactive compounds quickly [97]. Microwave-assisted alkali (8.16 M potassium hydroxide (KOH) at 60 °C) pretreatment provided a 3.25-fold higher yield of lutein from lyophilized marine microalgae *Chlorella sorokiniana* Shihira & Krauss in a short time of 1.47 min, compared to the conventional method (saponification for 30 min at 60 °C). In this study, the microscopic observations revealed the significant destruction of cellular structures and cell-wall components by microvalve-assisted alkali treatments. These observations suggested that microwave heating probably caused the vibration of water and other polar molecules, resulting in enormous pressure on the cell walls, leading to cell disruption and an enhanced mass transfer of lutein into the solvent.

A combination of cellulolytic and pectinolytic enzymes followed by ethyl acetate extraction provided the oleoresin with the highest amount of phenolic compound and the highest lycopene recovery (11.5 mg lycopene/g oleoresin), as well as improved antioxidant properties, from pretreatment of industrial tomato waste [102]. The enzymatic presentment conditions were optimized as: an enzymatic reaction temperature of 40 °C, an enzyme:enzyme ratio of 1, an enzyme:substrate ratio of 0.2 mL/g, a reaction time of 5 h, a solvent (ethyl acetate):substrate ratio of 5 mL/g, and an extraction time of 1 h.

Using the lyophilized biomass of various microalgal species, including fucoxanthin-rich *Isochrysis galbana* Parke, astaxanthin-rich *H. pluvialis*, and β-carotene-rich *Chlorella* sp. Beijerinck and *Scenedesmus almeriensis* nom. nud., different solvent combinations provided a different yield of the individual as well as the total CARs; however, in general, a tricomponent solution consisting of ethanol/hexane/water (77:17:6 *v*/*v*/*v*) provided the optimum yield [103]. 

## 6. Bioaccessibility and Bioavailability

The amount of bioactive and nutrient constituents that are solubilized for intestinal uptake (called the bioaccessible fraction) is much more crucial than the contents available in the food [104]. CARs are associated with proteins and other macromolecules within chromoplasts covered by rigid cell walls, which act as structural barriers for CAR release. Moreover, due to the lipid-soluble properties of CARs, the presence of lipids in a diet plays a vital role in bioaccessibility [105]. In addition, lipid digestion is affected by the microstructural properties of food, such as the presence of pectin, a primary dietary fiber in fruits and vegetables that influences CAR bioaccessibility [104,105].

Several factors have been investigated to enhance CARs’ micellization and bioaccessibility, including high-pressure homogenization (HPH) [104,106,107,108], a pulsed electric field (PEF) [109], low-temperature pasteurization (LP), and encapsulation [110]. Moreover, dietary phytochemicals, such as polyphenols, phytosterols, fatty acids, tocopherols, and divalent metals, may affect the uptake of CARs [107].

The rigid cell walls of microalgae act as a natural barrier to (lipophilic) nutrients during digestion. A substantially higher bioaccessibility of CARs (8–16%) and n-3 LC-PUFAs (27–29%) were observed for an HPH-treated (100 MPa) biomass of *Nannochloropsis* sp. Hibberd, compared to untreated biomass (1–6% for CARs and 13% for n-3 LC-PUFA) [108].

The molecular structure of CARs significantly influences their stability and bioavailability [33,111]. The astaxanthin-diesters with long-chain and saturated fatty acids (SFAs) showed higher thermal stability (at 60 °C) than astaxanthin monoester and free astaxanthin [111]. This suggests that an increase in the length of the carbon chain, a decrease in the unsaturation of fatty acids (as unsaturated fatty acids (UFAs) are more prone to oxidation, compared to SFAs), and an increase in the esterification degree of astaxanthin (diesters and monoesters) were beneficial to the thermal stability of astaxanthin. Interestingly it was also shown that the bioavailability of astaxanthin showed opposite trends to thermal stability, as the astaxanthin concentration in ICR mice serum revealed that astaxanthin-esters with short-chain fatty acids (SC-FAs) had higher bioavailability than long-chain fatty acids (LC-FAs), whereas astaxanthin-esters with high-UFAs had higher bioavailability than with SFAs [108]. This suggests that astaxanthin-esters with SC-FAs can be more easily digested into free-astaxanthin than astaxanthin with LC-FAs, as astaxanthin-esters probably hydrolyze to free-astaxanthin by cholesterol ester hydrolase (CEH) before being absorbed passively through the brush border of the enterocytes [112]. In contrast to the thermal stability investigated in this study, astaxanthin-monoesters showed a significantly higher bioavailability than astaxanthin-diesters. Among the all-molecular structure of astaxanthin investigated in this study, astaxanthin-DHA monoester showed the highest bioavailability.

(*Z*)- and (*E*)-configurations have also been shown to influence the bioavailability of CARs. The (all-*E*)-lycopene is more bioaccessible than (*Z*)-lycopene [36]. In the male rat model, astaxanthin (*Z*)-isomers, especially (13*Z*)-astaxanthin, have shown superior bioavailability and accumulation efficiency than the (all-*E*)-isomer [33]. In this study, in rats fed with (*Z*)-astaxanthin, the astaxanthin concentrations in the liver, kidney, lung, adrenal gland, prostate, testis, and skin were 4–37 times higher than in those fed (all-*E*)-astaxanthin.

Micro- and nanoencapsulation, as well as nanodispersion and nanoemulsion, are well known for enhancing the solubility of lipophilic CARs in aqueous solutions [36]. Moreover, they can facilitate bioaccessibility and bioavailability by enhancing stability throughout the gastrointestinal tract, assisting matrix release, improving micelle formation, and facilitating rapid transfer to the enterocytes [113,114,115]. An in vitro digestion model showed a higher (26%) release of astaxanthin from whey protein and gum arabic microcapsules compared to astaxanthin delivered in the form of oleoresin (14.6%) [113]. Similarly, in this study, the experiments on male BALB/c mice showed a 2-fold higher absorption from astaxanthin microcapsules than from astaxanthin oleoresin.

In mixed carrot, apple, and peach mixed juice, HPH substantially influenced water-soluble pectin’s characteristics (e.g., a decreased molecular weight and a branching and enhanced degree of methyl esterification), as well as lipid digestion and volume fraction of undigested particles, resulting in an enhanced bioavailability of CARs [104].

The daily intake of 500 mL of freshly squeezed orange juice or processed utilizing LP, HPP, or PEP treatment for 14 d in 12 healthy adults (six males and six females aged 20–32 years) showed a significant increase in serum β-cryptoxanthin concentration, with no statistically significant differences related to processing methods [109]. The authors suggested that the lack of a significant difference among the treatment groups was probably due to the high variability of the participants’ serum CAR concentrations. 

A particle-size reduction from HPH (150 MPa) was drastically responsible for an approximately 5-fold enhancement of the in vitro bioaccessibility of CARs in citrus juices compared to freshly prepared juice, suggesting that the particle size of the food matrix had a critical influence on the bioaccessibility of the CARs [106].

## 7. Encapsulation of CARs

The incorporation and delivery of CARs in food products are limited due to their poor water solubility and chemical instability. Encapsulation has been proved as the most suitable strategy to minimize these limitations [114,116]. CAR encapsulation into lipids (nanoliposomes, nanoemulsions, solid lipids, and nanostructured lipids), inorganic (gold nanoparticles, quantum dots, and carbon nanotubes), and polymeric nanoparticles (NPs) have been utilized to enhance the water solubility, storage stability, controlled and sustained release, bioaccessibility, bioavailability, and bioactivity of CARs [110,116,117,118,119]. In addition, natural small molecules (NSMs) are emerging as potential nanocarriers, as they can form self-assembled supramolecular nanostructures such as dehydrotrametenolic acid, betulinic acid, ursolic acid, and oleanolic acid [120,121]. For example, it was shown that a β-carotene/oleanolic acid NP emulsion displayed a substantially enhanced water dispersibility and an improved stability while also providing gastric protection and controlled release in simulated gastric fluid (SGF) and simulated intestinal fluid (SIF) [120]. In short, studies have shown that NSMs with inherent bioactivities exhibit health benefits [120], and have an excellent performance as drug carriers for photodynamic therapy [122] and drug delivery [121].

The polymeric NPs made from natural biodegradable polymers such as polysaccharides, alginate (ALG), chitosan (CS), and proteins [123] are widely utilized for CAR encapsulation. For example, astaxanthin-loaded CS oligosaccharide/ALG nanoparticles fabricated using oil-in-water emulsification followed by ionotropic gelation showed good stability during storage and exposure to oxidation, UV light, heat, acidic–alkaline, and simulated gastrointestinal fluid conditions, with enhanced bioaccessibility, bioavailability, and antioxidant activity [110]. As another example, a complex formed by fatty acids (FAs) and protein was also reported to be able to encapsulate CARs. In a study of fatty-acid-mediated protein (bovine serum albumin)–astaxanthin encapsulation, the FAs with longer carbon chain lengths (e.g., stearic acid, C18:0) or unsaturated double bonds (e.g., oleic acid, C18:1; linoleic acid, C18:2; or arachidonic acid C20:4) played a better role in improving the bioaccessibility of astaxanthin compared to lauric acid (C12:0) and palmitic acid (PA, C16:0) acid. Interestingly, in this study, DHA led to a sharp increase in the particle size and turbidity, and provided the lowest storage stability and bioaccessibility [124]. Another study showed that coencapsulated liposomes using hydrophilic (ascorbic acid) and hydrophobic (β-carotene) cavities showed improved encapsulation efficiency, free-radical-scavenging activities, and storage stability compared to a single component [125]. Finally, astaxanthin encapsulation in biopolymer-based NPs fabricated using a stearic acid–chitosan conjugate and sodium caseinate showed a good encapsulation capacity, with up to a 6% loading ratio [126]. Moreover, in this study, the aqueous dispersibility and bioactivity of encapsulated astaxanthin were greatly improved, confirmed by the in vitro antioxidant and antifibrogenic activities in LX-2 human hepatic stellate cells.

Currently, several microencapsulated CAR formulations are produced commercially [105]. LycoRed Ltd., Be’er Sheva, Israel, is producing and marketing microencapsulated lycopene (LycoBeads 5% and 20% alginate beadlets) for dietary supplements, which may be suitable for hard shell capsules and tablets (source: https://www.lycored.com/lycopene, accessed on 23 February 2022). In addition, microencapsulated beadlets containing (all-*E*)-lycopene and some (*Z*)-isomers are produced and marketed by BASF and Roche Vitamins commercially [105].

## 8. Normal, Safe, and Desirable Intake of CARs

No dietary reference intakes (DRIs) exist for CARs, especially for non-provitamin A CARs. Suggestions for their intake are primarily based on epidemiological studies. The practical dietary suggestion of CARs, recently reviewed by Bohm et al. [127], revealed that blood total carotenoid concentrations of <1000 nM/L can increase the risk of chronic diseases.

The DRI for vitamin A is 900 µg, which can be supplied by 10.8 mg/d of β-carotene, or 21.6 mg/d of α-carotene intake [127], as α-carotene has 50% provitamin activity compared to β-carotene. The β-carotene, α-carotene, β-cryptoxanthin, and other provitamin A CARs are the only source of vitamin A for vegans, as vegans’ diet lacks meat and milk, which can provide preformed vitamin A. For nonvegetarians, a meat-based diet can also supply preformed vitamin A to the body [128].

**Figure 5:**
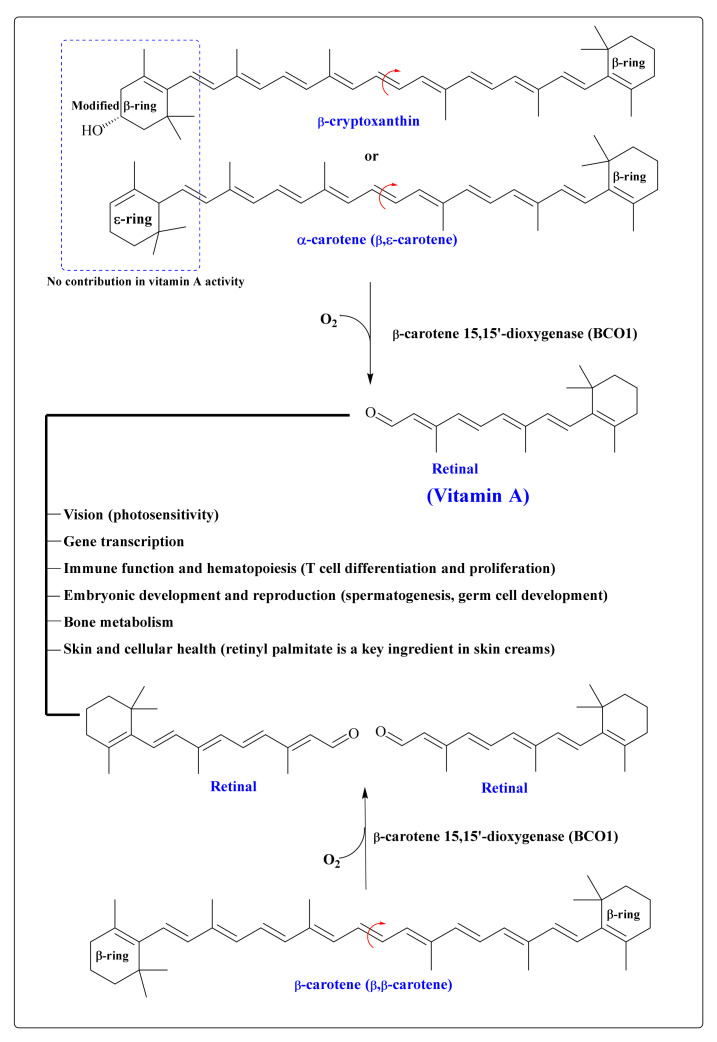
The presence of two unmodified β-ionone rings in one molecule of β-carotene can provide two molecules of retinol (vitamin A; 100% provitamin A activity), while α-carotene and β-cryptoxanthin contain only one β-ionone ring structure (50% provitamin A activity).

Carotenoid molecules with an unmodified β-ionone ring structure can be converted to vitamin A in the body. Due to the presence of two unmodified β-ionone rings in β-carotene, one molecule of β-carotene can provide two molecules of retinol (vitamin A; 100% provitamin A activity), while α- carotene and β-cryptoxanthin contain only one β-ionone ring structure (50% provitamin A activity) (Figure 5).

Beyond the rare exceptions, normal intake of CARs is considered safe [129]. The acceptable daily intakes (ADIs) of natural CARs and apo CARs, which are in the range of 14 (astaxanthin)–420 mg/d (bixin) for a person weighing 70 kg, is several times higher than the normal intakes (<10 mg/d) [129,130,131]. The ADI for β-carotene is 7–15 mg/d [129,132]. The National Health and Nutrition Examination Survey (NHANES; 2015–2016) in the United States suggested that among that country’s population, lycopene intake was most dominant (4.8 mg/d), followed by β-carotene (1.9 mg/d), lutein + zeaxanthin (1.4 mg/d), α-carotene (0.34 mg/d), and β-cryptoxanthin (0.086 mg/d) [49]. Moreover, in Europe, Australia, and other American countries, the dominance of lycopene, β-carotene, and lutein/zeaxanthin has been recorded [131].

Significant variability exists in the ADIs of CARs. For instance, the RDIs for astaxanthin range from 2–24 mg/d, with no safety concerns for natural astaxanthin supplementation at levels of 0.24 mg/d/kg body weight (BW) [133]. Surprisingly, some of these studies were performed using synthetic CARs [133]. In a review evaluating the ADI of astaxanthin, Brendler and Williamson [133] suggested that the recommended ADI should be based only on natural astaxanthin.

## 9. CARs in the Human Body

Although >40 different types of CARs are present in the human diet [25], only six CARs, namely lycopene, α- and β-carotene, lutein and zeaxanthin, and β-cryptoxanthin, represented >95% of the total CARs in the blood [134]. The serum CARs followed a similar trend to the dietary intake (except α-carotene) with the highest concentration of lycopene (0.804 µmol/L), followed by β-carotene (0.308 µmol/L), lutein + zeaxanthin (0.277 µmol/L), β-cryptoxanthin (0.202 µmol/L), and α-carotene (0.075 µmol/L) [49]. CARs are principally stored in adipose tissues and the liver [32]. CARs in the body vary considerably depending on geographical regions, smoking status, body mass index, and gender [135]. 

Despite the high dietary intake, some CARs are detected in small amounts in the plasma, rather than their hydrolyzed product being detected in a significant amount [136]. For instance, in a randomized intervention trial involving 22 healthy adults, administration of fucoxanthin-rich whole biomass of the microalgae *P. tricornutum* (5.3 g biomass containing 30 mg fucoxanthin/d for two weeks), only a small amount of fucoxanthin was recorded in plasma after the intervention, while fucoxanthinol (hydrolysis products of fucoxanthin) was detected in a significant amount after one and two weeks of intervention (232 and 482 nM/L, respectively) [137]. Moreover, after the intervention, a significant amount of amarouciaxanthin A (111 nM/L) was also recorded in the plasma, indicating the further metabolism of fucoxanthinol to amarouciaxanthin A. 

## 10. Health Benefits of CARs

**Figure 6:**
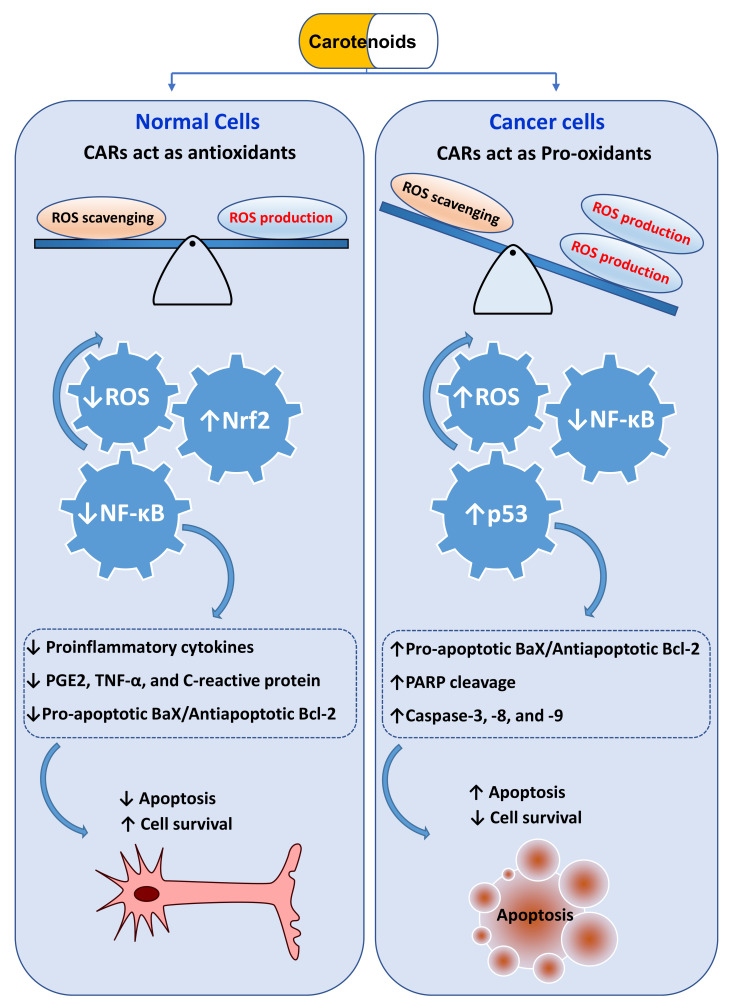
The antioxidant (in normal cells) and pro-oxidant properties of carotenoids regulate the reactive oxygen species (ROS) and modulate the nuclear factor kappa-light-chain-enhancer of activated B cells (NF-κB); nuclear factor-erythroid 2-related factor 2 (Nrf2), responsible for apoptosis of cancer cells; and survival of normal cells. Abbreviations: BAX, B-cell lymphoma 2 associated X; Bcl-2, B-cell lymphoma 2; PARP, poly (ADP-ribose) polymerase; PGE2, prostaglandin E2; TNF-α, tumor necrosis factor-alpha.

Attributable to the presence of high contents of antioxidant compounds, such as ascorbic acid, polyphenolic acids, tocopherols, and CARs in fruits, vegetables, and whole grains, their intakes minimize the risk of numerous chronic diseases, such as CVDs, neurodegenerative disorders, T2D, and various types of cancer [138,139]. In general, these chronic diseases are linked to increased levels of proinflammatory mediators, including oxidized phospholipids (e.g., OxLDL), circulating proinflammatory cytokines (e.g., interleukin (IL)-8, -6, and -1), inflammatory-stimulating prostaglandin E2 (PGE2), tumor necrosis factor-alpha (TNF-α,) nuclear factor kappa-light-chain-enhancer of activated B cells (NF-κB), and C-reactive protein (CRP). Owing to their antioxidant properties, CARs can regulate the levels of these mediators by oxidative stress modulation or by nuclear factor-erythroid 2-related factor 2 (Nrf2) and peroxisome proliferator-activated receptor (PPAR)-mediated overexpression of antioxidant and cytoprotective Phase II enzymes [131,140,141,142,143,144,145,146,147] (Figure 6). CAR-mediated Nrf2 signaling primarily plays a vital role in diminishing inflammatory responses and oxidative stress [143,146].

The markers of oxidative stress in epidemiological studies were recently reviewed by Bohn [146]. The European Food Safety Authority (EFSA) recommends F2-isoprostanes biomarkers for the direct measurements of lipid peroxides and oxLDL [146].

MetS, such as excess abdominal adiposity with hyperglycemia, elevated blood pressure, lower concentration of high-density lipoprotein cholesterol (HDL-c), and hypertriglyceridemia, lead to an increased risk of T2D and CVDs [148]. In a meta-analysis of 11 studies consisting of case-control, cross-sectional, longitudinal cohort, and randomized controlled trials (RCTs), an inverse relationship between metabolic syndrome and total CARs was found (odds ratio (OR) of 0.66). This inverse relationship was the strongest for β-carotene, followed by α-carotene and β-cryptoxanthin [148].

In an umbrella review of 17 articles with 20 health outcomes, tomato intake was inversely associated with coronary heart disease mortality, prostate and gastric cancer, cerebrovascular disease mortality, and all-cause mortality [4]. In addition, in this umbrella review, dietary lycopene intake or serum lycopene levels were inversely associated with prostate cancer, stroke, CVD, MetS, male infertility, and all-cause mortality. However, in this study, the authors suggested that the strength of evidence assessed using the Grading of Recommendation Assessment, Development and Evaluation (GRADE) framework was not high. 

A systematic review and meta-analysis of 26 RCTs investigating the effects of CARs on selected inflammatory parameters indicated a significant effect of CARs on alleviating CRP and IL-6, with a weighted mean difference (WMD) of −0.54 mg/L and −0.54 pg/mL, respectively; however, the effect on TNF-α was not significant [147]. In this study, individual CARs, astaxanthin, lutein/zeaxanthin, and β-cryptoxanthin also significantly decreased CRP levels. However, only lycopene (WMD: −1.08 pg/mL) led to a significant decrease in IL-6. In this study, the authors stressed that in the RCTs, the following parameters should be critically considered: (1) nutritional habits, controlling variations in weight, and lifestyle habits (e.g., physical activity and smoking); (2) evaluation of compliance rate through biomarkers for CARs consumption; and (3) restricting dietary intake of CARs.

The antioxidant activities of CARs are mostly investigated in in vitro experimental systems, where they have displayed powerful activities. However, only a few human studies are available, and the ability of CARs to alleviate oxidative stress in the body is inconclusive. For instance, astaxanthin is categorized as a potent antioxidant. However, in a meta-analysis of nine RCTs, astaxanthin showed only a borderline significant antioxidant effect between the control and intervention groups, with a malondialdehyde (MDA)-lowering effect for lipid peroxidation [149]. In this study, only astaxanthin doses of >20 mg/d showed a significant antioxidant effect on the total antioxidant capacity, superoxide dismutase, and isoprostane, while doses <20 mg/d showed no significant effect.

### 10.1. CARs Regulate PI3K/Akt/mTOR Signaling

**Figure 7:**
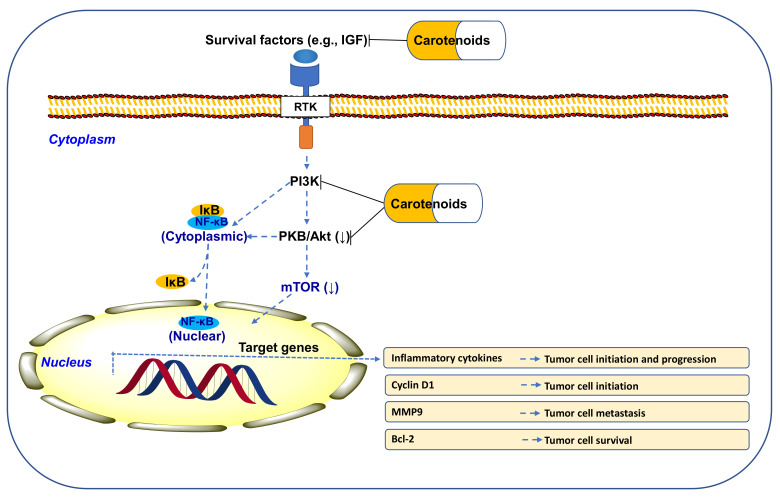
Carotenoids block the phosphoinositide 3-kinase (PI3K)/phosphorylated protein kinase B (PKB or Akt)/mechanistic target of rapamycin (mTOR) signaling pathways, thus reducing tumor cell initiation, progression, and metastasis. Abbreviations: Bcl-2: B-cell lymphoma 2: IGF: Insulin-like growth factor: MMP9: Matrix Metallopeptidase 9.

The phosphoinositide 3-kinase (PI3K)/phosphorylated protein kinase B (PKB or Akt)/mechanistic target of rapamycin (mTOR) signaling pathways play crucial roles in the maintenance of body homeostasis [150]. However, the aberrant activation of PI3K/Akt/mTOR signaling is closely correlated with cancer, T2D, CVDs, and neurodegenerative diseases [150]. Interestingly, on the other hand, enhanced PI3K/Akt/mTOR signaling is implicated in cellular and physiological regeneration and pathological conditions, including tissue injury or ischemia, neurodegeneration, and MetS-like insulin resistance [150]. CARs are well known to modulate PI3K/Akt/mTOR signaling positively and negatively [151], thus possessing a crucial clinical significance. For instance, nuclear translocation of an active NF-κB heterodimer leads to the activation of various target genes involved in antiapoptosis (e.g., B-cell lymphoma 2 (Bcl-2)), cell cycle (cyclin D1), prometastasis, and proinflammatory cytokines [11]. Carotenoids block these events, thus reducing tumor cell initiation, progression, and metastasis (Figure 7).

### 10.2. CARs Protect from Cancer

Several epidemiological, clinical, and preclinical studies have suggested the beneficial role of CARs in reducing cancer incidence and progression [49,139,152]. However, the recent expert report by the World Cancer Research Fund (WCRF)/American Institute for Cancer Research (AICR) concluded that evidence suggesting that consumption of food containing CARs reduces the risk of colon cancer is limited/no-conclusion [87]. However, in this report, the panel mentioned that there is evidence of a strong inverse relationship between CAR intake and ER-negative breast cancer risk.

Historically, the most influential dietary CAR intervention studies on men and women at high risk of developing lung cancer (e.g., asbestos-exposed and smokers), an α-tocopherol β-carotene cancer prevention trial (ATBC) [153] and a β-carotene and retinol efficacy trial (CARET) [154], showed consistent results of a higher incidence of lung cancer (relative risk (RR) of >1.1) among the active intervention groups. Interestingly, in the CARET study, at the baseline, an inverse association was recorded between β-carotene dietary intake estimates and serum levels, and later lung cancer incidence. β-Carotene (30 mg/d) and vitamin A (25000 IU retinyl palmitate) supplementation in CARET raised the serum β-carotene levels 12-fold from the baseline. It is possible that such levels (nearly 10–20 times higher than normal intake) are toxic or cause disequilibrium with other components important to redox homeostasis. These studies suggested that β-carotene may act as a pro-oxidant under high ROS levels and high solute concentrations in lungs of smokers, and highlighted the need to better understand how CARs behave in varied cellular conditions.

A review of 6 cohort, 11 case-control, 3 cross-sectional, and 2 controlled clinical trials of the influence of CARs on prostate cancer occurrence suggested that increased consumption of CARs, especially lycopene from tomatoes, might be related to a reduced risk of developing prostate cancer [155]. For instance, among the European and American populations, intake of >10 mg/d of lycopene is related to a reduced risk of diagnosed prostate cancer by at least 10%, compared to an intake of <3.6 mg/d.

In the nested case-control study, data from the nurses’ health studies (NHS and NHSII) comprising 1919 cases and 1695 controls showed that higher levels of circulating CARs provided greater protection to the women at high risk (due to genetic predispositions or high mammographic density (MD)) for breast cancer [156]. In this study, the highest quartile of plasma CARs (≥142.1 μg/dL) had significant absolute risk reductions (ARR; 28.6%) compared to those in the lowest quartile of CARs (<84.6 μg/dL). Similarly, among the women with a high MD (≥50%), the highest quartile of plasma CARs was associated with a 37.1% ARR when comparing the lowest quartiles.

A meta-analysis of case-control and cohort studies investigating the relation between CAR intake or circulating CAR concentrations and bladder cancer risk in men and women, comprising 22 studies involving 516,740 adults, showed that dietary β-cryptoxanthin intake and circulating concentrations of lutein/zeaxanthin, β-carotene, and α-carotene were inversely associated with bladder cancer risk. In this study, bladder cancer risk decreased by 42% for every 1 mg increase in daily intake of dietary β-cryptoxanthin, while a 1 µmol/L increase in the circulating concentration of α-carotene, lutein/zeaxanthin, and β-carotene reduced bladder cancer risk by 76, 56, and 27%, respectively.

In a systematic review and meta-analysis of one clinical trial, one pooled study, and eight cohorts, comprising 19,450 breast cancer cases, β-carotene intake was significantly associated with higher breast cancer survival (RR of 0.70). At the same time, no significant benefits were recorded from the intake of other non-provitamin A CARs (e.g., lycopene and lutein), retinol, and provitamins A CARs (e.g., α-carotene and β-cryptoxanthin) [157].

CARs are well known for their potent antioxidant function in the cellular system. However, in cancer cells with an innately high level of intracellular ROS due to rapid metabolism and higher lipid peroxidation, lower levels of antioxidant enzymes, and the higher laves of reactive metals (e.g., Fe (III) and Cu(II), CARs, including astaxanthin, fucoxanthin, β-carotene, and lycopene, may act as potent pro-oxidant molecules and trigger ROS-mediated apoptosis [41]. In our recent studies, β-cryptoxanthin from mandarin oranges (*C. unshiu* Marc.) [158] and lutein from marigold (*Tagetes erecta* L.) petals [159] upregulated ROS generation, with a concordant enhanced expression of caspase-3, B-cell lymphoma 2 associated X (Bax), and p-53 mRNA, and suppression of antiapoptotic Bcl-2 in human cervical carcinoma (HeLa) cells. These events triggered nuclear condensation, a substantial loss of mitochondrial membrane potential, activation of caspase-3 proteins (studied in β-cryptoxanthin traded cells), and finally, cleavage of nuclei DNA of HeLa cells.

CARs may behave differently with varying cellular levels. In our study, astaxanthin at 20–40 µmol/L concentrations triggered the apoptosis of astroglioma multiforme (GBM) U251-MG cells [160]. However, in this study, 4–8 µmol/L of astaxanthin upregulated the proliferative cell cycle by suppressing the expression of tumor protein p53, cyclin-dependent kinase (Cdk) 2, and p-Cdk2/3 protein levels. These results suggested that astaxanthin had a hormetic effect on the astroglioma U251-MG cells. Thus, dose levels and cellular uptake should be critically considered, as they might have consequences opposite to those expected.

The tumor necrosis factor (TNF)-related apoptosis-inducing ligand (TRAIL) is known to trigger apoptosis in various cancer cells; thus, TRAIL-based anticancer drugs are under trials [161]. Our recent study on four types of GBM cells expressing low (U251-MG and T98-MG) and high (CRT-MG and U87-MG) levels of superoxide dismutase (SOD) suggested that astaxanthin sensitizes low-SOD2-expressing GBM cells to TRAIL treatment by pathways involving mitochondrial potential-mediated apoptosis that SOD2 can inhibit. These observations suggested that astaxanthin might be effective in GBM treatment only under specific conditions of low SOD2 activity. In addition, astaxanthin usage and effects should be critically monitored in GBM patients due to the higher overall SOD2 expression in GBM tumors.

### 10.3. CARs in Obesity and T2D

Preclinical, clinical, and epidemiological studies have indicated the beneficial effects of CAR intake on obesity and associated pathophysiological disorders, including low-grade inflammation, hepatic steatosis, and insulin resistance [162]. The protective effects of lycopene against obesity are mediated via a downregulation of proinflammatory mediators (e.g., IL-1 and -6, TNF-α, inducible nitric oxide synthase (iNOS), cyclooxygenase-2 (COX-2), matrix metalloproteinase-3 (MMP-3), and -9, and NF-κB), upregulation of anti-inflammatory mediators (IL-1 and transforming growth factor-β (TGF-β)), redox homeostasis, browning of white adipose tissue (WAT), enhanced lipolysis and lipogenesis, and decreased insulin resistance [8].

Sirtuin 1 (SIRT1) is a key protein involved in increased insulin sensitivity and other critical functions related to cellular regulation. Thus, it has been suggested that improving insulin resistance and SIRT1 expression can reverse vascular aging [163]. Lycopene (100 µmol/L) has been shown to promote the proliferation and migration of human umbilical vein endothelial cells (HUVECs) by upregulating the SIRT1 protein [163]. In this study, in rats, 100 mg/kg of lycopene supplementation for eight weeks reversed aging; ameliorated insulin resistance; improved vascular aging in the thoracic aorta; increased muscle capillary density (improving energy metabolism); and upregulated the expression of Akt (roles in glucose metabolism), glucose transporter type 4 (Glut4; insulin-regulated glucose transporter), and vascular endothelial growth factor (VEGF; regulator of angiogenesis) [163].

Gut dysbiosis and inflammation are two crucial characteristics of obesity and related diseases [164]. CARs can mediate the composition of gut microbiota to alleviate obesity. In HFD-male C57BL/6J mice, 0.1% fucoxanthin supplementation (% diet weight, *w*/*w*) from brown kelp (seaweed) *U. pinnatifida* reversed HFD-induced gut microbiota dysbiosis by suppressing the growth of obesity-/inflammation-related *Erysipelotrichaceae* and *Lachnospiraceae* while promoting the growth of *Bifidobacterium* Orla-Jensen, *Lactobacillus* Beijerinck/*Lactococcus* Schleifer, and some butyrate (a short-chain fatty acid (SCFA))-producing bacteria [164].

A recent double-blind RCT on 45 middle-aged obese (BMI: 30–45 kg/m^2^; aged 45–65 y) individuals from Iran showed that lutein (from marigold petals) supplementation (20 mg/d for 10 weeks; n = 23) in combination with a low-calorie diet (LCD; 25% less than the energy requirements) could improve body composition and lipid profile compared to placebo (n = 22), who received only an LCD [165]. In this study, those given lutein supplementation significantly experienced more body (mean difference of −1.76%) and visceral fat loss (mean difference of −0.82%) compared to placebo (mean difference of −0.82 and −0.31%, respectively). 

A meta-analysis of seven RCTs and eight observational studies revealed that low levels of serum CARs are a risk factor for overweight and obesity [166]. Moreover, in this meta-analysis, CAR supplementation was significantly associated with bodyweight reductions, a decrease in body mass index, and waist circumference losses.

Data from two separate cohorts studied in Sydney, Australia, between 2008 and 2013 consisting of nonobese (n = 14); and obese (n = 66; BMI ≥ 30 kg/m^2^) male and female subjects using serum and adipose tissue showed that adipose tissue had ζ-carotene, phytoene, and phytofluene stored in substantial amounts, accounting for 25% of the total adipose tissue CARs [167]. In this study, α-, β-, and ζ-carotene and retinol correlated inversely with adiposity and insulin resistance in the liver (*p* ≤ 0.028) and adipose tissue (*p* = 0.023), but not muscle insulin resistance, suggesting that these CARs played an insulin-sensitizing role locally in the liver and adipose tissue tissues.

The expression of some miRNAs, such as miRNA-146a, is upregulated in diabetic and hyperglycemic patients, and is involved in regulating inflammatory markers, including NF-κB [168]. In a randomized, double-blind, placebo-controlled clinical trial in 44 patients with T2D receiving 8 mg/d of oral astaxanthin (n = 22) or placebo (n = 22) for eight weeks, significantly decreased plasma levels of MDA and IL-6 and expression of miR-146a were found, probably due to a reduction in the ROS levels [168].

### 10.4. CARs in Cardiovascular Diseases (CVDs)

Hypertension is considered a leading risk factor for stroke, heart attacks, renal failure, and many other complications [169]. Epidemiological studies have revealed positive associations between a higher intake or status of CARs and a lower risk of CVDs [169,170].

A meta-analysis of RCTs, consisting of 10 studies, revealed that lycopene supplementation considerably decreased systolic blood pressure (SBP), especially among the participants with a baseline SBP ≥ 130 mmHg, with a lycopene intake of ≥15 mg/d for ≥8 weeks [169]. In this study, the diastolic blood pressure (DBP) was also found to have decreased significantly among the hypertensive subjects (DBP of >80 mmHg).

Another meta-analysis of 25 studies, mainly from the USA (15) and Finland (8), including 211,704 participants, showed that individuals with the highest serum concentration (0.41 µmol/L) or that were in the highest consumption category of lycopene (9.81 mg/d) had substantially lower risks of stroke (hazard ratio (HR) 0.74) and CVDs (HR 0.86) [170]. Moreover, in this study, a significantly lower risk of mortality (HR 0.63) was recorded among the individuals categorized with the highest serum concentration of lycopene.

### 10.5. CARs in Osteoporosis and Muscle Strength

Excess oxidative stress inhibits bone formation and enhances bone resorption, leading to higher bone loss and an increased risk of osteoporosis [171]. However, the antioxidant properties of CARs may help in improving bone health. Cross-sectional data from the 2005–2018 NHANES on individuals with an average age of 61.9 years (57.5% female) and valid data on CAR intake and bone mineral density (BMD) showed a lower risk of osteoporosis among the individuals with a high intake of β-carotene and β-cryptoxanthin [171]. In this study, a very high intake of lutein/zeaxanthin was also associated marginally but significantly with a lower risk of osteoporosis.

Age-related declines in muscle mass and strength (relegated mostly to a lack of exercise and dietary protein, or vitamin D) were associated with declines in physical function and other complications [172]. A diet rich in antioxidants, such as CARs, may attenuate the age-related loss of muscle and physical function. In a prospective cohort study, among the elder participants (average age of 61 years) in the Framingham offspring study, higher intakes of total CARs, lutein/zeaxanthin, and lycopene were associated with an increased annualized change in grip strength and a faster gait speed [172].

### 10.6. CARs in Neurodegenerative Disease and Mental Health

The antioxidant and anti-inflammatory activities of CARs protect against cognitive decline and neurodegenerative diseases, such as Alzheimer’s disease (AD) and Parkinson’s disease (PD) [173,174,175]. The data from 2011–2014 NHANES participants (n = 2796, ≥60 years), a cross-sectional survey, suggested that the highest quartile of lutein/zeaxanthin intake of 1.2 mg/d (estimated from two nonconsecutive 24 h diet recalls) was associated with cognitive improvement (a 2.52-point increase on the digit symbol score test), compared with the lowest quartile (0.3 mg/d).

In a prospective cohort study comprising 1580 mother–child pairs, a higher maternal lutein/zeaxanthin intake (daily mean of 2.6 mg) during the first and second trimesters of pregnancies was associated with an improved behavior-regulation ability and verbal intelligence in midchildhood [176]. 

A pooled meta-analysis of nine RCTs with a total of 4402 nondemented subjects (age ranging from 45 to 78 years) revealed a significant beneficial effect of CAR intervention on cognitive functions (Hedge’s g = 0.14) [177].

A high dietary CAR intake was associated with cognitive performance in an older population [178]. Data from the 2014 Health and Retirement Study, a nationally representative panel study of older US adults (≥50 years), and the 2013 Health Care and Nutrition Study, which assessed dietary intake via the Harvard food frequency questionnaire (FFQ) in a subsample of respondents, suggested that older adults in the highest quartiles for lutein/zeaxanthin intake (5.46 mg/d) had significantly higher immediate word recall and delayed word recall scores than those in the lowest quartile (0.74 mg/d) [178]. This study suggested that leafy vegetables, dark yellow vegetables, cruciferous vegetables, seafood, legumes, eggs, and fruit may serve as a significant and vital predictor of dietary lutein/zeaxanthin intake.

In a community-based cohort of 927 older (average age of 81 years) Midwestern US residents with an average follow-up of 7 years, a higher intake (assessed using FFQ) of total CARs (in particular lutein/zeaxanthin) was associated with a substantially lower hazard of AD, possibly through inhibition of brain β-amyloid deposition and fibril formation [173]. In this study, when comparing the top (median intake: 24.8 mg/d) and bottom quintiles (median intake: 6.7 mg/d) of total CARs, the multivariate HR (95% CI) was 0.52 (48% reduction in the rate of AD).

Elevated levels of oxidative stress and neuronal apoptosis play crucial roles in traumatic brain injury. Astaxanthin treatments significantly enhanced the expression of Nrf2, peroxiredoxin 2 (Prx2; ROS scavenger), and SIRT1 (regulator of the Nrf2 signaling) proteins, while it downregulated the expression of phosphorylated apoptosis signal-regulating kinase 1 (p-ASK1) and p-p38 proteins in male C57BL/6 mice [179]. In this study, with Nrf2 knockout or inhibition of Prx 2, SIRT 1 alleviated the beneficial effects of astaxanthin after traumatic brain injury. These observations indicated that astaxanthin ameliorated oxidative damage and neuronal apoptosis via SIRT1/Nrf2/Prx2/ASK1/p38 signaling.

In the male albino rats, 10 mg/kg BW lycopene treatments for 21 d have shown to alleviate the acrylamide induced toxicity by reversing the decline in the hematological parameters (white blood cells, red blood cells, and lymphocytes counts, hematocrit value, and hemoglobin concentration), brain neurotransmitters concentrations (serotonin and dopamine) and acetylcholinesterase (AChE) activity, as well as improved the levels of antioxidant (reduced glutathione and glutathione peroxidase (GPx)) and suppressed oxidative stress (MDA, nitric oxide, and protein carbonyl) biomarkers [180]. 

Alpha-synuclein (SNCA), a vital component of Lewy inclusion bodies (a neuropathological hallmark of PD and other disorders), is considered a key causative gene in the inception of familial PD [181]. MiR-7 microRNAs play a key regulatory role in the cellular system, and their levels are generally decreased in PD [181]. Astaxanthin treatments have shown to protect against endoplasmic reticulum (ER) stress and protect against PD-caused neuron damage by targeting miR-7/SNCA [181]. In SH-SY5Y cells, 5–50 µmol/L astaxanthin treatments significantly reversed 1-methyl-4-phenylpyridinium (MPP+)-induced cell-viability inhibition and apoptosis promotion by inhibiting ER stress, and also abolished the downregulation of miR-7 and upregulation of SNCA protein expression mediated by MPP+.

Central insulin resistance mediated by serine phosphorylation of insulin receptor substrate-1 (IRS-1) has been shown to play a critical role in AD [182]. Moreover, glycogen synthase kinase-3β (GSK-3β) activity is increased in AD, which leads to hyperphosphorylation of the tau protein, resulting in the formation of neurofibrillary tangles. In an amyloid-β (1–42) peptide-induced AD model in Wistar rats, 0.5–1 mg/kg of oral astaxanthin treatments for 28 days reversed cognitive and memory impairments dose-dependently, as assessed by the novel object recognition test and the Morris water maze test. In this study, ASX attenuated oxidative stress; IRS-S307 and glycogen synthase kinase-3β (GSK-3β) activities; and levels of soluble Aβ (1–42), TNF-α, AChE, and nitrite in the hippocampus.

Crocin, a significant CAR in saffron, showed potential therapeutic effects against lipopolysaccharide (LPS)-induced neuroinflammation and depressive-like behaviors in Kunming mice [183]. In this study, 20–40 mg/kg BW of crocin inhibited the LPS-induced expression of IL-1β, IL-18, and TNF-α in the hippocampi of LPS-injected mice. Moreover, crocin alleviated the LPS-induced expression of NF-Κb, p65, nucleotide-binding domain leucine-rich repeat with a pyrin-domain containing protein 3 (NLRP3), and caspase-1 in the hippocampus.

### 10.7. Eye and Skin Health

In humans, the xanthophylls lutein, zeaxanthin, and meso-zeaxanthin (3R,3’S-zeaxanthin: a metabolic product of lutein in the body) accumulate in the fovea and inner plexiform layer of the retina as macular pigment, which is responsible for the protection of the retinal membrane against harmful effects of short-wavelength high-intensity light, and improves visual acuity [42,184]. Interestingly, it has been shown that low-density lipoproteins (LDLs) and HDLs mediate the selective uptake of zeaxanthin and lutein in the human retina [128]. Epidemiological and clinical studies have witnessed the vital role of dietary lutein/zeaxanthin in reducing the risk of age-related macular degeneration (AMD) [184].

CARs are widely used in cosmeceuticals, mostly due to their UV-protection activities. Moreover, CARs may improve skin characteristics [185,186]. A systematic review of 11 clinical studies revealed that 3 to 6 mg/d of astaxanthin supplementation for 2 to 16 weeks improved skin texture, appearance (wrinkles), and moisture content [187].

### 10.8. Other Benefits

Free astaxanthin and encapsulated astaxanthin (20 µmol/L) have shown antifibrogenic (prevention of fibrotic scarring) activities in LX-2 cells by significantly lowering the transforming growth factor β1 (TGFβ1)-induced fibrogenic gene (actin alpha 2 (ACTA2) and collagen, type i, alpha 1 (COL1A1) mRNA levels, as well as alpha-smooth muscle actin (α-SMA) and COL1A1 protein levels [126].

Aquaporins (AQPs) regulate the osmotic gradient in cells, which plays a significant role in maintaining the water and ionic homeostasis in living cells [188]. The changes in AQP expression in the kidney can lead to nephrogenic diabetes insipidus and nephrotoxicity [188]. In Kunming mice, 5 mg/kg of lycopene supplementation had protective effects against atrazine-induced nephrotoxicity by maintaining ionic homeostasis, reversing the changes in Ca^2+^–Mg^2+^–ATPase activity controlling the expression of AQPs (especially AQP2) on the cell membrane [188].

Lycopene treatment (5 mg/kg BW/d) has been shown to alleviate di(2-ethylhexyl) phthalate (DEHP; a chemical pollutant)-induced caspase-1-dependent pyroptosis and the inflammatory response in the spleens of mice. In this study, lycopene treatments inhibited the expression of caspase-1-activating proteins, as well as inflammatory factors such as NF-κB, IL-1β, and IL-18.

## 11. Agroindustrial Waste Valorization, Biorefinery, and Circular Bioeconomy Perspective

Domestic and industrial processing of fruits and vegetables generates considerable waste and byproducts. For instance, the citrus juice and tomato-canning industries generate a significant amount of wastes that are rich in CARs [85,189,190,191,192,193] and several other bioactive antioxidants [194] with health-promoting potentials [195,196,197,198]. The disposal of such wastes is a huge environmental concern. However, fruit and vegetable waste valorization to recover the economically vital compounds can solve this issue. Moreover, utilization of waste and byproducts can create a surplus revenue that can substantially improve fruit and vegetable processing economics. Much research has been conducted to recover commercially vital CARs from fruit and vegetable wastes [192,199]. The extraction assisted with ultrasound [189,200] may result in a good amount of CAR recovery from the CAR-rich food waste. Moreover, modern extraction methods that use greener solvents (supercritical CO_2_) for extracting CARs can play an important role in contributing to a cleaner environment and sustainable food production [192,199,201].

Microalgae are a rich source of nutritionally vital CARs, lipids, bioactive peptides, and many other economically and industrially vital compounds [46,202]. Microalgal lipids are rich in vital nutritional LC n-3 PUFAs such as DHA and EPA [64]. Moreover, biofuel production from microalgal lipids has recently attracted considerable interest globally [202]. Cultivation and downstream processing may account for 50–90% of the total expenditure in producing microalgae-based CARs [68]. This can be mitigated by simultaneous extraction of CARs, lipids, bioactive peptides, and other valuable compounds from microalgal biomass. In addition, agroindustrial wastes, such as molasses, can be utilized as low-cost carbon sources for the fermentation process to produce CARs and other economically and industrially vital compounds [47]. Moreover, microalgae-based biorefineries can be considered as a potential solution toward sustainability by utilizing advanced extraction techniques that integrate the coproduction of nutritionally vital compounds with bioethanol and biodiesel [68,202,203]. The economic evaluation of the sustainable production process of CARs, glycerol, polar lipids, and proteins from *D. salina* (biomass feed of 84 m^3^/year) suggested profitability under the best scenario, with a reasonable payback period of just 1.1 years [203]. 

The halophilic microalgae species, including *D. salina,* required 3–5 M salinity in the medium to grow and produce β-carotene [204]. From a circular bioeconomy perspective, the reuse of seawater reverse osmosis (SWRO) brine to produce β-carotene from *D. salina* was successfully investigated [204]. According to an estimate, 750 tons of algal β-carotene can be produced daily by utilizing SWRO brine [204]. Moreover, agroindustrial and industrial wastes such as sugarcane molasses can be recycled to supply the nutrient requirements for microalgal and yeast cultivation [205,206].

## 12. Conclusions and Future Prospective

CARs are fascinating molecules with significantly diverse chemical structures, such as a varied length of the polyene chain, the presence of diverse functional groups and their positioning (e.g., α and β), glycosylation of a hydroxyl group, esterification with various fatty acids, acyclic and cyclic structure, and (*E*)- or (*Z*)-configuration. These chemically diverse structures substantially influence the physical and biological properties of CARs.

Given the growing demand and market potential for CARs, alternate solutions such as biofortification of cereal crops and microbial production of CARs need to be developed by leveraging advanced synthetic biology tools. Future research should focus on integrating multiomic analyses such as transcriptomics, proteomics, and metabolomics in order to understand the various bottlenecks involved in the transgenic production of CARs. Further, large-scale industrial production of microbial CARs requires the optimization of the fermentation process by using low-cost agroindustrial waste.

Apart from provitamin A activity, antioxidant activities are primarily responsible for the most biological function of CARs. The antioxidant activities of CARs regulate oxidative stress, responsible for the suppressed expression of proinflammatory mediators and overexpression of antioxidant and cytoprotective Phase II enzymes. These events reduce the risk of metabolic syndromes (CVDs and T2D), cancer, neurodegenerative diseases, and several other chronic and inflationary diseases, as supported by several mechanistic and epidemiological studies. 

However, some epidemiological studies showed no association/effect between CAR intake and the incidence of these diseases. The following interpretation can be drawn from these inconsistent findings: (1) the health benefits of bioactive compounds, including those of CARs on the human body, may not be visible during a short study period. However, they can contribute throughout life as part of the daily diet [67]; (2) compared to a single compound, the cumulative effects of several bioactive foods are more effective in the reduction of the risk of chronic diseases. For instance, compared to isolated lycopene, consumption of whole fruit was considered more beneficial in CVDs [207]; (3) the substantially high dose of β-carotene (30 mg/d) and vitamin A (25000 IU retinyl palmitate) that was used in CARET raised the serum β-carotene levels 12-fold from the baseline. It is possible that such levels are toxic, or at least cause serious disequilibrium with other compounds essential to redox relationships; (4) interindividual differences in genes related to C absorption and metabolism may substantially modify the responses (CVs >70%) to the supplemented CAR [11,112,127]. For example, a single-nucleotide polymorphism (SNP) in ELOVL fatty acid elongase 2, β-carotene oxygenase 1 (BCO1), and the scavenger receptor class B member 1 (SCARB1) are associated with a substantial variation in lycopene, lutein, and β-carotene bioavailability [105,208]. In addition, intestine-specific homeobox (ISX) transcription factor (induced by vitamin A) has been shown to repress the expression of SCARB1 and BCO1, thus preventing β-carotene uptake via a negative feedback mechanism [209]. Moreover, lifestyle habits (e.g., smoking and alcohol consumption) may influence the outcome of epidemiological studies.

Presently, some limitations are associated with the use of CARs as a functional food. Table 1 summarizes the possible solution to such limitations.

## Figures and Tables

**Table 1:** The limitations associated with the use of CARs as functional food, and possible solutions to such limitations.

Limitations	Possible Solution	Reference
Low contents of CARs in staple foods crops	Biofortification of crops	[89,90,91,92]
Solvent residues in extracted CARs	Green-solvent-assisted extraction	[199]
Water insolubility, low storage stability, bioaccessibility, bioavailability, and bioactivity of CARs	Encapsulation of CARs	[110,117,120,207]
Low bioaccessibility and bioavailability	Mechanical processing of foods	[104]
Food waste (e.g., fruit peel) contains more CARs than edible parts, or loss of CARs in food waste	Waste valorization for recovery of CARs	[199]
High cost of microbial CAR production	Use of a biorefinery and a circular bioeconomy approach	[202,204,205,206,210]

## Abbreviations

AChE	Acetylcholinesterase
AD	Alzheimer’s disease
ADIs	Acceptable daily intakes
ALG	Alginate
AMD	Age-related macular degeneration
AQPs	Aquaporins
ASK1	Apoptosis signal-regulating kinase 1
ATBC	α-Tocopherol β-Carotene Cancer Prevention Trial
Bax	B-cell lymphoma 2 associated X
Bcl-2	B-cell lymphoma 2
BCO1	β-carotene oxygenase 1
BHA	Butylated hydroxyanisole
BHT	Butylated hydroxytoluene
BMD	Bone mineral density
BW	Body weight
CARET	β-Carotene and Retinol Efficacy Trial
CARs	Carotenoids
Cas9	CRISPR-associated protein 9
Cdk	Cyclin-dependent kinase
CEH	Cholesterol ester hydrolase
COL1A1	Collagen, type i, alpha 1
COX-2	Cyclooxygenase-2
CRISPR	Clustered regularly interspaced short palindromic repeats
CRP	C-reactive protein
CS	Chitosan
CVDs	Cardiovascular diseases
DBP	Diastolic blood pressure
DCW	Dry cell weight
DHA	Docosahexaenoic acids
DMAPP	Dimethylallyl pyrophosphate
DRIs	Dietary reference intakes
DW	Dry weight
EPA	Eicosapentaenoic acid
FFQ	Food Frequency Questionnaire
FW	Fresh weight
GBM	Astroglioma multiforme
Glut4	Glucose transporter type 4
GM	Genetic modification
GPx	Glutathione peroxidase
GRADE	Grading of Recommendation Assessment, Development and Evaluation
GSK-3β	Glycogen synthase kinase-3β
HDL-c	High-density lipoprotein cholesterol
HeLa	Human cervical carcinoma
HPH	High-pressure homogenization
HR	Hazard ratio
HUVECs	Human umbilical vein endothelial cells
IGF	Insulin-like growth factor
IL	Interleukin
iNOS	Inducible nitric oxide synthase ()
IPP	Isopentenyl pyrophosphate
IRS-1	Insulin receptor substrate-1
LC-PUFAs	Long-chain-polyunsaturated fatty acids
LP	Low-temperature pasteurization
MAE	Microwave-assisted extraction
MD	Mammographic density
MDA	Malondialdehyde
MEP	2-C-methyld-erythritol 4-phosphate
MetS	Metabolic syndromes
MMP	Matrix metallopeptidase
mTOR	Mechanistic target of rapamycin
MVA	Mevalonate
n-3	Omega-3
NF-κB	Nuclear factor kappa-light-chain-enhancer of activated B cells
NPs	Nanoparticles
Nrf2	Nuclear factor-erythroid 2-related factor 2
NSMs	Natural small molecules
OxLDL	Oxidized phospholipids
PARP	Poly (ADP-ribose) polymerase
PD	Parkinson’s disease
PEF	Pulsed electric field
PGE2	Prostaglandin E2
PI3K	Phosphoinositide 3-kinase
PKB	Phosphorylated protein kinase B
PPARs	Peroxisome proliferator-activated receptor
Prx2	Peroxiredoxin 2
RCTs	Randomized controlled trials
ROS	Reactive oxygen species
SBP	Systolic blood pressure
SCARB1	Scavenger receptor class b member 1
SCE	Supercritical CO_2_ extraction
SC-FAs	Short-chain fatty acids
SGF	Simulated gastric fluid
SIF	Simulated intestinal fluid
SIRT1	Sirtuin 1
SNCA	Alpha-synuclein
SNP	Single-nucleotide polymorphism
SOD	Superoxide dismutase
SWRO	Seawater reverse osmosis
T2D	Type 2 diabetes
TGF-β	Transforming growth factor-β
TNF-α	Tumor necrosis factor-alpha
TRAIL	Tumor necrosis factor (TNF)-related apoptosis-inducing ligand
UAE	Ultrasonication-assisted extraction
UFAs	Unsaturated fatty acids
VEGF	Vascular endothelial growth factor
WAT	White adipose tissue
WMD	Weighted mean difference
α-SMA	Alpha-smooth muscle actin
